# Association of triglyceride-glucose index and its combinations with sarcopenia among community-dwelling older adults: based on the Chongqing Aging and Sarcopenia Evaluation (CHASE) cohort

**DOI:** 10.3389/fphys.2025.1595517

**Published:** 2025-05-15

**Authors:** Xinyu Yu, Siqi Jiang, Zhiyu Chen, Keke Ren, Shan Li, Yetao Luo, Deqing Chen, Qinghua Zhao, Huanhuan Huang

**Affiliations:** ^1^ Department of Nursing, The First Affiliated Hospital of Chongqing Medical University, Chongqing, China; ^2^ Department of Orthopedics, The First Affiliated Hospital of Chongqing Medical University, Chongqing, China; ^3^ Department of Nosocomial Infection Control, The Second Affiliated Hospital of Army Medical University, Chongqing, China; ^4^ Department of Endocrinology, The People’s Hospital of Rongchang District, Chongqing, China

**Keywords:** sarcopenia, triglyceride glucose, triglyceride glucose-body mass index, older adult, insulin resistance

## Abstract

**Background:**

The triglyceride-glucose (TyG) index serves as an alternative index for assessing insulin resistance (IR). The relationship between the TyG index and its combined indicators and sarcopenia remains insufficiently explored.

**Aim:**

To investigate the association between the TyG index and its combined parameters and sarcopenia.

**Methods:**

This cross-sectional study encompassed 792 community-dwelling older adults from the Chongqing Aging and Sarcopenia Evaluation (CHASE) cohort. The multivariate logistic regression model was used to analyze the relationship between the TyG index and its combined parameters, which include the triglyceride glucose-body mass index (TyG-BMI), triglyceride glucose-calf girth (TyG-CG), triglyceride glucose-waist circumference (TyG-WC), and triglyceride glucose-waist-to-hip ratio (TyG-WHR). The receiver operator characteristic (ROC) curve was utilized to assess the diagnostic effect of each index. The integrated discrimination improvement index (IDI) and net reclassification improvement (NRI) were applied to compare the diagnostic efficacy among the indices.

**Results:**

The TyG index and its combined parameters demonstrated a significant correlation with the risk of sarcopenia (*P* < 0.05). The area under the ROC curve (AUC) for the TyG index in predicting the risk of sarcopenia was 0.623 (95% confidence interval: 0.570–0.675). Notably, composite parameters incorporating the TyG index showed enhanced predictive performance. Specifically, TyG-BMI showing the highest AUC of 0.892 (95% confidence interval: 0.860–0.924), indicating its strong predictive potential. Furthermore, the diagnostic efficacy of TyG-BMI was superior to that of all other indicators in both the IDI and NRI.

**Conclusion:**

The TyG index demonstrates diagnostic potential for sarcopenia identification, with significantly enhanced accuracy when combined with other parameters. Among them, TyG-BMI is a robust sarcopenia risk predictor due to its superior predictive power.

## 1 Introduction

Sarcopenia is an age-related, progressive, and generalized loss of skeletal muscle mass and muscle strength or physical function (Cho et al., 2022; Chen et al., 2020), which was first proposed by Irwin Rosenberg in 1989 (Rosenberg, 2011), and officially confirmed as a muscle disease in 2016 (Cao and Morley, 2016). According to reports, muscle mass and strength in the legs of people over the age of 50 will decline by 1%–2% and 1.5%–5% per year (Keller and Engelhardt, 2013), and the cross-sectional area of the vastus lateralis is reduced by up to 40% between the ages of 20 and 80 years (Kalyani et al., 2014). It is reported that the prevalence of sarcopenia among older adults is relatively high (Xin et al., 2021), the prevalence rates for individuals aged 60 and above range from 10% to 27% (Petermann-Rocha et al., 2022), affecting 11%–50% of those aged 80 and above (Cruz-Jentoft et al., 2014). Estimates suggesting that the condition will affect approximately 500 million individuals by 2050 (Liu et al., 2021). Sarcopenia is associated with an increased risk of adverse health outcomes in older adults, including falls, mobility limitations, hip fractures, and mortality (Cawthon et al., 2020). However, the diagnosis of sarcopenia is constrained by several factors, including the lack of clinical popularization, the high cost of instruments, and the absence of standardized diagnostic protocols, the diagnosis is limited and not suitable for large-scale epidemiological investigation, which will further lead to the risk of sarcopenia cannot be better identified (Cruz-Jentoft and Sayer, 2019; Ooi and Welch, 2024). Consequently, the identification of sarcopenia remains a challenge due to the screening tool, particularly in community settings (Sayer and Cruz-Jentoft, 2022).

As a multifactorial disease, sarcopenia is influenced by risk factors, genetic factors, and lifestyles (Aslam et al., 2023). It is well established that insulin resistance (IR) is pathophysiological linkage with sarcopenia (Hou et al., 2024). The skeletal muscle is the principal tissue of insulin-induced glucose metabolism (Kopecky et al., 2024). The loss of skeletal muscle will lead to impaired muscle contraction function and abnormal metabolism of the body (Nishikawa et al., 2021). Impairment of insulin stimulation disrupts systemic glucose homeostasis damage and lead to IR (Cleasby et al., 2016). Hyperinsulinemia caused by the occurrence of IR will accelerate the degradation of protein, slow down the protein synthesis, and increase the amount of myostatin to cause the decrease of skeletal muscle, thus causing the occurrence or aggravation of sarcopenia (Liu and Zhu, 2023). Therefore, evaluating IR levels could be clinically valuable approach for predicting the risk of sarcopenia. The triglyceride-glucose (TyG) index serves as a simple surrogate marker for IR based on fasting blood glucose and triglyceride (Pan Y. et al., 2024). Its use for assessing IR in a broad population using the TyG index is advantageous and convenient, as it obviating the need for insulin quantification (Tajima et al., 2024).

Previous studies have shown that the TyG index and its combined parameters can effectively predict the risk of sarcopenia (Zhang et al., 2024). However, the measurement of calf girth (CG) is significant for the screening of sarcopenia, but few studies have focused on the composite indicator triglyceride glucose-calf girth (TyG-CG). Additionally, most existing studies have included middle-aged (Kim et al., 2021) and younger (Kwon et al., 2024) populations, with few studies exclusively focusing on older adults. Therefore, our study specifically targets community-dwelling older adults aged 65 and above. In addition to commonly studied composite indicators, we incorporate TyG-CG to thoroughly investigate the correlation between the TyG index and its associated parameters with sarcopenia, thereby aiming to establish a novel reference index for risk screening of sarcopenia among older adults.

## 2 Materials and methods

### 2.1 Study design

This cross-sectional study utilizes data from the Chongqing Aging and Sarcopenia Evaluation (CHASE) cohort. The CHASE is a prospective cohort study based on the National Basic Public Health Service Project in Chongqing. It focuses on older adults in the community and encompasses the collection of personal information, physical examination, and management of health records to assess health status (Jiang et al., 2024).

### 2.2 Inclusion and exclusion criteria

In this study, the inclusion criteria were as follows: (a) age ≥65 years; (b) provision of informed consent; (c) availability of complete research data. The exclusion criteria were: (a) participants with metallic implants in the body, such as cardiac pacemakers and steel nail plates; (b) participants with cognitive impairment, severe mental illness, and daily living ability disorders who were unable to complete the test; (c) participants with clinically visible edema; (d) participants in the end stage of disease or acute exacerbation of chronic disease; (e) participants were unable to walk and go out independently.

### 2.3 Sample size

In our previous study, the prevalence of sarcopenia among community-dwelling older adults was found to be 17.5% (Jiang et al., 2024), set Z = 1.96 (α = 0.05), the allowable error d = 0.05, the expected sample size was calculated as 222. Considering a 10%–20% follow-up loss rate, the minimum sample size was about 267 people.

### 2.4 Data collection

General information was collected using a self-made questionnaire that included questions on gender, age, educational level, marital status, occupational status, medical insurance, smoking, drinking, hypertension, and diabetes. All participants were subjected to a physical examination. The data collected included waist circumference (WC, cm), calf girth (CG, cm), body mass index (BMI, kg/m^2^), waist-to-hip ratio (WHR), and BMI was calculated as weight/height^2^. In addition, blood biochemical tests were performed on all participants, assessing a total of fifteen indicators, including lymphocyte count (x10^9^L), neutrophil count (x10^9^L), hemoglobin (HGB, g/L), platelet count (x10^9^L), serum alanine transaminase (ALT, U/L), serum glutamic oxaloacetic transaminase (AST, U/L), total protein (TP, g/L), albumin (ALB, g/L), globulin (GLB, g/L), serum creatinine (Scr, μ mol/L), fasting plasma glucose (FPG, mg/dL), total cholesterol (TC, mg/dL), triglyceride (TG, mg/dL), low-density lipoprotein (LDL, mg/dL) and high-density lipoprotein (HDL, mg/dL). In this study, the TyG index and its combinations were calculated as follows:

(a) TyG = ln [fasting triglyceride (mg/dL) × fasting plasma glucose (mg/dL)/2]
(b) TyG-WC = TyG/WC
(c) TyG-BMI = TyG/(weight/height^2^) = TyG/BMI
(d) TyG-WHR = TyG/(waist circumference/hip circumference) = TyG/WHR
(e) TyG-CG = TyG/CG

### 2.5 Diagnosis of sarcopenia

Sarcopenia is a chronic disease characterized by a progressive decline in muscle mass and muscle strength. In this study, the AWGS 2019 diagnostic criteria were used (Chen et al., 2020), which include:(a) Handgrip strength was measured, and the diagnostic threshold was <28.0 kg for males and <18.0 kg for females; (b) Bio-impedance analysis (BIA) was used to test skeletal muscle index (SMI), and the diagnostic cut-off value was <7.0 kg/m^2^ for males and <5.7 kg/m^2^ for females. Participants were diagnosed with sarcopenia when satisfying both (a) and (b). Handgrip strength was assessed using an electronic hand dynamometer (Xiangshan, EH101, China). Participants performed two trials of maximal grip effort with their dominant hand, and the maximum value was recorded in kilograms (kg). SMI was measured using a bioelectrical impedance analysis device (InBody, 270, South Korea).

### 2.6 Data analysis

SAS 9.4 software (Copyright^©^ 2016 SAS Institute Inc. Cary, NC, United States) was performed for statistical analysis. The measurement data conforming to normal distribution were described by mean plus or minus standard deviation (mean ± SD), and an independent sample t-test was used for comparison between groups. The skewed distribution of measurement data was presented by the median and interquartile range [M (P25, P75)], and the Mann-Whitney U test was used for comparison between groups. The count data were statistically described by frequency and composition ratio, and the chi-square test was used for inter-group comparison. The multivariate logistic regression model explored the relationship between the TyG index and its combined parameters and sarcopenia. In the model, we sequentially adjusted for general demographic factors, including age, gender, and education level, as well as clinical data such as lymphocyte count, neutrophil count, and HGB levels. These adjustment factors encompass both external social factors and internal physiological data of the participants, thereby comprehensively excluding the influence of confounding variables on the study outcomes. The receiver operator characteristic (ROC) curve was used to assess the diagnostic effect of the TyG index and its combined parameters in differentiating sarcopenia. The accuracy, sensitivity, and specificity were calculated after internal validation by leave-one-out cross-validation. The diagnostic effects of different indexes were compared by the integrated discrimination improvement index (IDI) and the net reclassification improvement (NRI). All *P* < 0.05 were considered statistically significant.

## 3 Results

### 3.1 Baseline characteristics

A total of 792 older adults were included in this study, which met the minimum sample size requirement. The average age of the participants was 71.71 ± 5.77 years, with 44.32% males and 55.68% females. Most of them had junior high school education (41.79%), married (77.65%), and retired (91.41%). Compared with non-sarcopenia group, the sarcopenia group was found to be older and less educated (as shown in Table 1), and HGB, ALT, ALB, BMI, CG, WC, and WHR were lower than those in the non-sarcopenia group, while FPG, TG, TyG, TyG-BMI, TyG-CG, TyG-WC, and TyG-WHR were higher (*P* < 0.05). As shown in Table 2.

**TABLE 1:** Comparison of general information.

Variables	Total (n = 792)	Non-sarcopenia (n = 643)	Sarcopenia (n = 149)	t/χ^2^ value	P value
Age (years)	71.71 ± 5.77	71.08 ± 5.24	74.43 ± 7.06	−5.448	<0.001
Gender					
Female	441 (55.68)	359 (55.83)	82 (55.03)	0.031	0.860
Male	351 (44.32)	284 (44.17)	67 (44.97)		
Educational level					
Illiterate	91 (11.49)	69 (10.73)	22 (14.77)	9.514	0.049
Elementary school	116 (14.65)	85 (13.22)	31 (20.81)		
Junior high school	331 (41.79)	279 (43.39)	52 (34.90)		
Senior high school	148 (18.69)	120 (18.66)	28 (18.79)		
College diploma or above	106 (13.38)	90 (14.00)	16 (10.74)		
Marital status					
Married	615 (77.65)	506 (78.69)	109 (73.15)	8.048	0.045
Divorced	13 (1.64)	12 (1.87)	1 (0.67)		
Widowed	111 (14.02)	80 (12.44)	31 (20.81)		
Other/prefer not to answer	53 (6.69)	45 (7.00)	8 (5.37)		
Vocational status					
Retired	724 (91.41)	589 (91.60)	135 (90.60)	0.153	0.695
Engaged in farming/work	68 (8.59)	54 (8.40)	14 (9.40)		
Medical insurance					
Resident medical insurance	121 (15.28)	92 (14.31)	29 (19.46)	7.911	0.019
Employee medical insurance	613 (77.40)	510 (79.32)	103 (69.13)		
Others	58 (7.32)	41 (6.38)	17 (11.41)		
Smoke					
No	692 (87.37)	560 (87.09)	132 (88.59)	0.246	0.620
Yes	100 (12.63)	83 (12.91)	17 (11.41)		
Drink					
No	703 (88.76)	565 (87.87)	138 (92.62)	2.734	0.098
Yes	89 (11.24)	78 (12.13)	11 (7.38)		
Hypertension					
No	293 (36.99)	235 (36.55)	58 (38.93)	0.294	0.588
Yes	499 (63.01)	408 (63.45)	91 (61.07)		
Diabetes					
No	523 (66.04)	425 (66.10)	98 (65.77)	0.006	0.940
Yes	269 (33.96)	218 (33.90)	51 (34.23)		

**TABLE 2:** Evaluation of clinical data.

Variables	Total (n = 792)	Non-sarcopenia (n = 643)	Sarcopenia (n = 149)	t/Z value	P value
Lymphocyte count (x10^9^L)	1.65 ± 0.49	1.66 ± 0.49	1.62 ± 0.48	0.911	0.363
Neutrophil count (x10^9^L)	3.41 ± 1.35	3.42 ± 1.41	3.36 ± 1.10	0.568	0.571
HGB (g/L)	138.93 ± 15.93	139.94 ± 15.83	134.57 ± 15.70	3.738	<0.001
Platelet count (x10^9^L)	181.00 (152.00, 217.00)	182.00 (153.00, 216.00)	178.00 (142.00, 222.00)	−0.497	0.619
ALT (U/L)	20.00 (16.00, 27.00)	20.00 (16.00, 27.00)	17.00 (14.00, 26.00)	−3.701	<0.001
AST (U/L)	20.00 (17.00, 24.00)	20.00 (17.00, 24.00)	20.00 (16.00, 25.00)	0.094	0.925
TP (g/L)	72.02 ± 5.54	72.00 ± 5.27	72.15 ± 6.59	−0.264	0.792
ALB (g/L)	43.76 ± 4.22	43.94 ± 4.02	42.98 ± 4.96	2.199	0.029
GLB (g/L)	28.33 ± 5.37	28.14 ± 5.05	29.14 ± 6.52	−1.750	0.082
Scr(μmol/L)	70.20 ± 22.97	69.94 ± 19.93	71.34 ± 33.11	−0.496	0.621
BMI (kg/m^2^)	24.46 ± 3.32	25.24 ± 2.91	21.10 ± 2.82	15.706	<0.001
CG (cm)	34.00 ± 3.45	34.42 ± 3.28	32.21 ± 3.60	7.268	<0.001
WC (cm)	84.41 ± 9.85	86.19 ± 9.28	76.74 ± 8.52	11.367	<0.001
WHR	0.89 ± 0.06	0.89 ± 0.06	0.85 ± 0.05	8.388	<0.001
FPG (mg/dL)	103.14 (92.16, 120.60)	101.70 (91.26, 117.36)	114.84 (97.20, 138.60)	5.478	<0.001
TC (mg/dL)	189.95 ± 42.96	188.57 ± 41.79	195.92 ± 47.39	−1.744	0.083
TG (mg/dL)	119.61 (85.06, 173.66)	117.84 (83.28, 161.25)	147.08 (93.03, 245.42)	4.696	<0.001
LDL (mg/dL)	111.56 ± 36.30	111.90 ± 34.72	110.06 ± 42.52	0.492	0.623
HDL (mg/dL)	50.35 ± 13.06	50.37 ± 12.59	50.24 ± 14.96	0.098	0.922
TyG	9.21 ± 0.35	9.18 ± 0.32	9.36 ± 0.41	−5.017	<0.001
TyG-BMI	0.38 ± 0.06	0.37 ± 0.04	0.45 ± 0.05	−17.531	<0.001
TyG-CG	0.27 ± 0.03	0.27 ± 0.03	0.29 ± 0.03	−9.861	<0.001
TyG-WC	0.11 ± 0.02	0.11 ± 0.02	0.12 ± 0.01	−11.495	<0.001
TyG-WHR	10.45 ± 0.81	10.32 ± 0.76	11.04 ± 0.72	−10.538	<0.001

### 3.2 The correlation between the TyG index and its combined parameters and sarcopenia

In the crude model, the result of multivariate logistic regression showed that there was a positive correlation between the TyG index, TyG-CG, TyG-WC, TyG-BMI, TyG-WHR, and sarcopenia (*P* < 0.001). After adjusting for all covariates in Model 3, TyG (OR = 3.943, 95% CI = 2.269–6.851), TyG-CG (OR = 1.478, 95% CI = 1.383–1.580), TyG-WC (OR = 1.403, 95% CI = 1.291–1.525), TyG-BMI (OR = 2.107, 95% CI = 1.791–2.479) and TyG-WHR (OR = 3.326, 95% CI = 2.527–4.379) were still positively correlated with sarcopenia (*P* < 0.001). The detailed results are presented in Table 3.

**TABLE 3:** Univariable and multivariable logistic analysis of the triglyceride-glucose (TyG) index and its combined parameters and Sarcopenia.

Variables	Crude model		Model 1^a^		Model 2^b^		Model 3^c^	
	OR (95% CI)	P value	OR (95% CI)	P value	OR (95% CI)	P value	OR (95% CI)	P value
TyG	4.283 (2.562, 7.161)	<0.001	3.615 (2.126, 6.149)	<0.001	3.782 (2.210, 6.472)	<0.001	3.943 (2.269, 6.851)	<0.001
TyG-CG	1.475 (1.385, 1.571)	<0.001	1.480 (1.386, 1.580)	<0.001	1.477 (1.384, 1.577)	<0.001	1.478 (1.383, 1.580)	<0.001
TyG-WC	1.392 (1.290, 1.503)	<0.001	1.393 (1.286, 1.509)	<0.001	1.404 (1.294, 1.523)	<0.001	1.403 (1.291, 1.525)	<0.001
TyG-BMI	2.147 (1.837, 2.508)	<0.001	2.126 (1.813, 2.492)	<0.001	2.146 (1.827, 2.521)	<0.001	2.107 (1.791, 2.479)	<0.001
TyG-WHR	3.325 (2.566, 4.309)	<0.001	3.240 (2.487, 4.221)	<0.001	3.255 (2.494, 4.248)	<0.001	3.326 (2.527, 4.379)	<0.001

^a^ Adjusted for age, gender, educational level, marital status, vocational status, and medical insurance.

^b^ Adjusted for smoke, drink, hypertension, and diabetes based on model 1.

^c^ Adjusted for lymphocyte count, neutrophil count, HGB, platelet count, ALT, AST, TP, ALB, GLB, and Scr based on model.

### 3.3 The accuracy of the TyG index and its combined parameters in predicting sarcopenia

**FIGURE 1:**
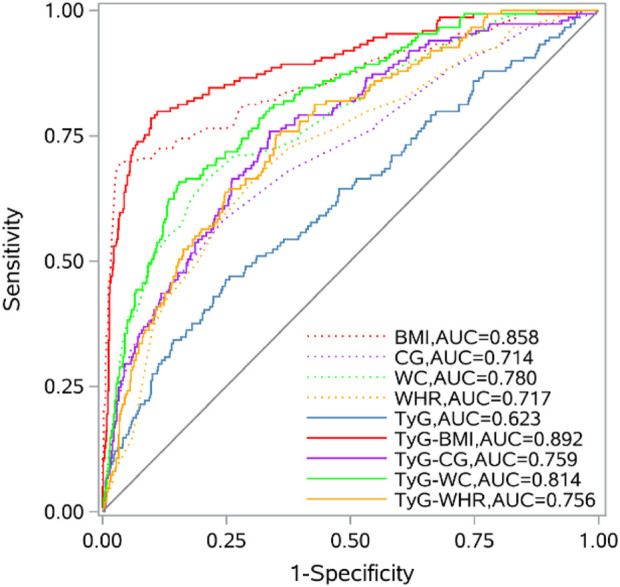
ROC curves of TyG and its combined parameters and biochemical indexes.

The ROC curve results indicated that BMI, CG, WC, WHR, FPG, TG, TyG, and its combined parameters all had certain diagnostic abilities for sarcopenia. Compared with the TyG index, the AUC of its combinations increased (*P* < 0.05). In detail, TyG-BMI (increased AUC = 0.269, 95% CI = 0.206, 0.333), TyG-CG (increased AUC = 0.137, 95% CI = 0.076, 0.197), TyG-WC (increased AUC = 0.191, 95% CI = 0.131, 0.252), TyG-WHR (increased AUC = 0.133, 95% CI = 0.081, 0.185). The AUC values of TyG-BMI, TyG-CG, TyG-WC, and TyG-WHR for sarcopenia were found to be higher than those of BMI, CG, WC, and WHR (*P* < 0.05). Among them, TyG-BMI had the highest AUC and the strongest predictive ability. Details are shown in Figure 1 and Table 4.

**TABLE 4:** ROC curve results and diagnostic effect.

Variables	Cut-off value	AUC (95% CI)	P value	Accuracy^a^	Sensitivity^a^	Specificity^a^
BMI (kg/m^2^)	21.20	0.858 (0.819, 0.898)	<0.001	91.29%	69.80%	96.27%
Calf girth (cm)	32.00	0.714 (0.666, 0.763)	<0.001	72.22%	58.39%	75.43%
WC (cm)	79.00	0.780 (0.737, 0.822)	<0.001	76.39%	66.44%	78.69%
WHR	0.86	0.717 (0.671, 0.763)	<0.001	68.69%	66.44%	69.21%
FPG (mg/dL)	107.82	0.644 (0.592, 0.695)	<0.001	63.13%	60.40%	63.76%
TC (mg/dL)	256.38	0.537 (0.484, 0.589)	0.169	79.55%	13.42%	94.87%
TG (mg/dL)	193.15	0.623 (0.568, 0.679)	<0.001	76.64%	42.28%	84.60%
LDL (mg/dL)	56.84	0.521 (0.468, 0.574)	0.444	79.55%	10.74%	95.49%
HDL (mg/dL)	46.40	0.518 (0.466, 0.570)	0.504	57.07%	46.31%	59.57%
TyG	9.36	0.623 (0.570, 0.675)	<0.001	69.70%	46.31%	75.12%
TyG-BMI	0.42	0.892 (0.860, 0.924)	<0.001	87.25%	79.87%	88.96%
TyG-CG	0.28	0.759 (0.717, 0.802)	<0.001	68.06%	75.84%	66.25%
TyG-WC	0.12	0.814 (0.776, 0.851)	<0.001	81.06%	65.77%	84.60%
TyG-WHR	10.54	0.756 (0.714, 0.797)	<0.001	66.92%	75.17%	65.01%

^a^ Leave-one-out cross-validation results.

Furthermore, the results of the IDI showed that the ability of TyG-BMI to diagnose sarcopenia increased by 29.5%, 23.5%, and 29.4%, respectively, compared with TyG-CG, TyG-WC and TyG-WHR (*P* < 0.05). The results of the NRI index showed that compared with TyG-CG, TyG-WC, and TyG-WHR, the possibility of TyG-BMI correctly judging sarcopenia increased by 112.7%, 110.9%, and 115.1% (P < 0.05). The related results are shown in Table 5.

**TABLE 5:** Comparison of diagnostic effects of different indicators.

Variables	Difference in AUC (95% CI)	P value	IDI (95% CI)	P value	NRI (95% CI)	P value
TyG-BMI vs. TyG	0.269 (0.206, 0.333)	<0.001	0.378 (0.325, 0.432)	<0.001	1.141 (0.992, 1.291)	<0.001
TyG-CG vs. TyG	0.137 (0.076, 0.197)	<0.001	0.083 (0.052, 0.114)	<0.001	0.603 (0.433, 0.774)	<0.001
TyG-WC vs. TyG	0.191 (0.131, 0.252)	<0.001	0.143 (0.107, 0.179)	<0.001	0.694 (0.527, 0.862)	<0.001
TyG-WHR vs. TyG	0.133 (0.081, 0.185)	<0.001	0.084 (0.058, 0.111)	<0.001	0.721 (0.555, 0.888)	<0.001
TyG-BMI vs. BMI	0.034 (0.017, 0.050)	<0.001	0.057 (0.037, 0.076)	<0.001	0.604 (0.439, 0.769)	<0.001
TyG-CG vs. CG	0.045 (0.020, 0.071)	0.001	0.036 (0.023, 0.049)	<0.001	0.425 (0.249, 0.600)	<0.001
TyG-WC vs. WC	0.034 (0.016, 0.053)	<0.001	0.019 (0.006, 0.033)	0.005	0.149 (-0.027, 0.326)	0.099
TyG-WHR vs. WHR	0.038 (0.008, 0.069)	0.013	0.040 (0.022, 0.058)	<0.001	0.361 (0.185, 0.538)	<0.001
TyG-BMI vs. TyG-CG	0.133 (0.093, 0.172)	<0.001	0.295 (0.255, 0.336)	<0.001	1.127 (0.978, 1.276)	<0.001
TyG-BMI vs. TyG-WC	0.078 (0.044, 0.112)	<0.001	0.235 (0.196, 0.275)	<0.001	1.109 (0.958, 1.260)	<0.001
TyG-BMI vs. TyG-WHR	0.136 (0.097, 0.176)	<0.001	0.294 (0.251, 0.337)	<0.001	1.151 (1.004, 1.297)	<0.001

## 4 Discussion

This study is the first to investigate the correlation between the TyG index and its combined parameters and sarcopenia in older adults in Chinese communities. Our findings indicate that the prevalence of sarcopenia was 18.81%. The multivariate logistic regression model demonstrated a correlation between the TyG index and its combined parameters with sarcopenia, and this correlation was not affected by confounding factors. The ROC curve indicated that the TyG index and its combined parameters possess diagnostic value for sarcopenia. Moreover, we employed the IDI and NRI for diagnostic effect comparisons. All results consistently demonstrated that TyG-BMI was the most predictive and diagnostically accurate indicator.

The TyG index may possess the ability to predict the risk of sarcopenia, as suggested by previous research. Yang et al. (2024) discovered a more pronounced correlation between the TyG index and sarcopenia through analysis of the NHANES database, and this correlation remained robust even after using logistic regression and adjusting for all relevant covariates, a finding that aligns with the results of our study. At the same time, the findings of our study reveal that TyG-BMI is the strongest predictor of the risk of sarcopenia, which is consistent with the results of Pan R. et al. (2024). However, this study focuses on older adults aged 65 and above, while Pan focuses on people aged 20–59, which also reflects from another perspective that TyG-BMI has the best predictive ability for sarcopenia at all ages. A cross-sectional study from South Korea reported a significant correlation between an elevated TyG index and low muscle mass (LMM) (Kim et al., 2021), which refines the research on sarcopenia screening indicators, and this represents an aspect that was not covered in our study. However, Xue (Wei and Liu, 2024) further highlighted the correlation between LMM and low muscle strength (LMS) with the TyG index and their results showed that LMM and LMS were positively and independently correlated with the TyG index, with more significant findings when both were present. In addition, sarcopenic obesity, which is characterized by the coexistence of sarcopenia and obesity with an increased BMI as one of the screening indicators, has been correlated with the TyG index in several studies, and the results showed that the TyG index could be a potential indicator of sarcopenic obesity (Kim et al., 2023). It is worth noting that studies on the TyG index and its combined parameters in relation to sarcopenia in China predominantly utilize the China Health and Retirement Longitudinal Study (CHARLS). For instance, Zhang et al. (2024) reported a negative correlation between the TyG index and its combined parameters and the risk of sarcopenia, which contradicts our findings. This discrepancy may stem from differences in population selection and muscle mass measurement methods. Zhang’s study used 2015 data from CHARLS, while our study focuses on community-dwelling older adults with more recent data collection. Additionally, they employed anthropometric equations for muscle mass measurement, whereas we used the BIA method recommended by AWGS 2019. Consequently, more field survey data are needed to focus on the association between the TyG index, its combined parameters and sarcopenia.

The mechanism underlying the relationship between the TyG index and sarcopenia remains not fully elucidated. Prior research indicates that IR is significantly associated with sarcopenia (Chen et al., 2023; Abbatecola et al., 2011), which is a trigger for sarcopenia and a result of sarcopenia (Li et al., 2024). In contemporary clinical practice, the hyperinsulinemic euglycemic clamp (HEC) and the homeostasis model assessment of insulin resistance (HOMA-IR) are the most commonly used methods to assess individual IR (Zhan et al., 2024). However, HEC is invasive and is only applicable in clinical settings. HOMA-IR requires the measurement of fasting insulin levels, making it less suitable for primary healthcare settings (Tahapary et al., 2022). The TyG index has the characteristics of high availability and convenience. It has become a robust choice for assessing IR (Simental-Mendía et al., 2008) and current evidence supports that the TyG index outperforms HOMA-IR in predictive capacity (Son et al., 2022), making it more amenable to primary healthcare work and large-scale epidemiological investigation (Sánchez-García et al., 2020). The results of this study not only demonstrate that the TyG index can effectively predict the occurrence of sarcopenia but also highlight the high predictive capacity of TyG-BMI. This composite biomarker, which combines the TyG index and BMI, is cost-effective and more effectively identifies IR than other alternative biomarkers, exhibiting superior detection power (Lim et al., 2019; Er et al., 2016). At present, the TyG index and its combined parameters had been employed to evaluate the risk of various chronic diseases, such as cardiovascular disease (Azarboo et al., 2024; Liu et al., 2023; Liang et al., 2023; Liu et al., 2022), peripheral artery disease (Samavarchitehrani et al., 2024), obstructive sleep apnea (Behnoush et al., 2024), stroke (Yang et al., 2023), cognitive impairment (Hao et al., 2024) and even dementia (Hong et al., 2021). In addition, research indicates that the perioperative may affect muscle function changes and increase the risk of surgical complications (Petra et al., 2025). Timely assessment of the TyG index and its related parameters during this period might effectively reduce the incidence of complications.

In summary, the current findings demonstrated that the TyG index and its combined parameters may have applications in primary medical and epidemiological investigation. Calculating and assessing them during health checkups or disease screenings can enhance predictive efficiency for sarcopenia and promote preventive and therapeutic efforts. This may help reduce the incidence of sarcopenia and improve overall quality of life.

This study possesses several notable strengths. First of all, this study conducted a field survey of community-dwelling older adults, capturing data that fully reflects the prevalence of sarcopenia and overall health in this demographic; secondly, this study adjusted the influence of confounding factors on the outcome to the maximum extent, and gradually adjusted the existing covariates; finally, the study employed multiple methods to investigate the predictive capacity of the TyG index and its combined parameters for sarcopenia risk, and conducted comparisons of their predictive abilities. It is noteworthy that our study population represents a special cohort. As one of the largest municipalities in China, Chongqing has unique characteristics of older adults, and its special diet structure and activity habits also make it unique. Thus, our research holds specific regional significance. However, this study also has several limitations. Firstly, due to the practical limitations of data collection, we did not collect data on physical function tests from the participants. Secondly, we did not exclude patients with diabetes but instead adjusted for it as a confounding factor, which is a problem worthy of further investigation in the future. Additionally, although we adjusted for potential confounding factors to the extent possible, we did not account for medication use in this elderly population. Finally, as a cross-sectional study, our study limits the deep exploration into causal relationships, necessitating further research and investigation.

## 5 Conclusion

Our study shows that there is a significant correlation between the TyG index and its combined parameters and sarcopenia, with TyG-BMI exhibiting the strongest predictive ability for the risk of sarcopenia. Consequently, TyG-BMI could be employed for screening and conducting large-scale epidemiological investigations of sarcopenia at the community health grassroots level. This approach would strengthen the early detection and intervention of sarcopenia, prevent and control the occurrence of the disease, delay its development, and ultimately improve the quality of life for older adults.

## 6 Core tip

The correlation between sarcopenia and insulin resistance (IR) has been confirmed. As a simple surrogate marker for IR, the association between the triglyceride-glucose (TyG) index and its combined parameters with sarcopenia remains unclear. This study is based on the Chongqing Aging and Sarcopenia Evaluation (CHASE) cohort, and the results show a significant correlation between the TyG index and sarcopenia, with its combined parameters further enhancing diagnostic accuracy. Among them, TyG-BMI demonstrated the highest diagnostic efficacy and superior predictive power for risk.

## Data Availability

The original contributions presented in the study are included in the article/Supplementary Material. Further inquiries can be directed to the corresponding authors.
